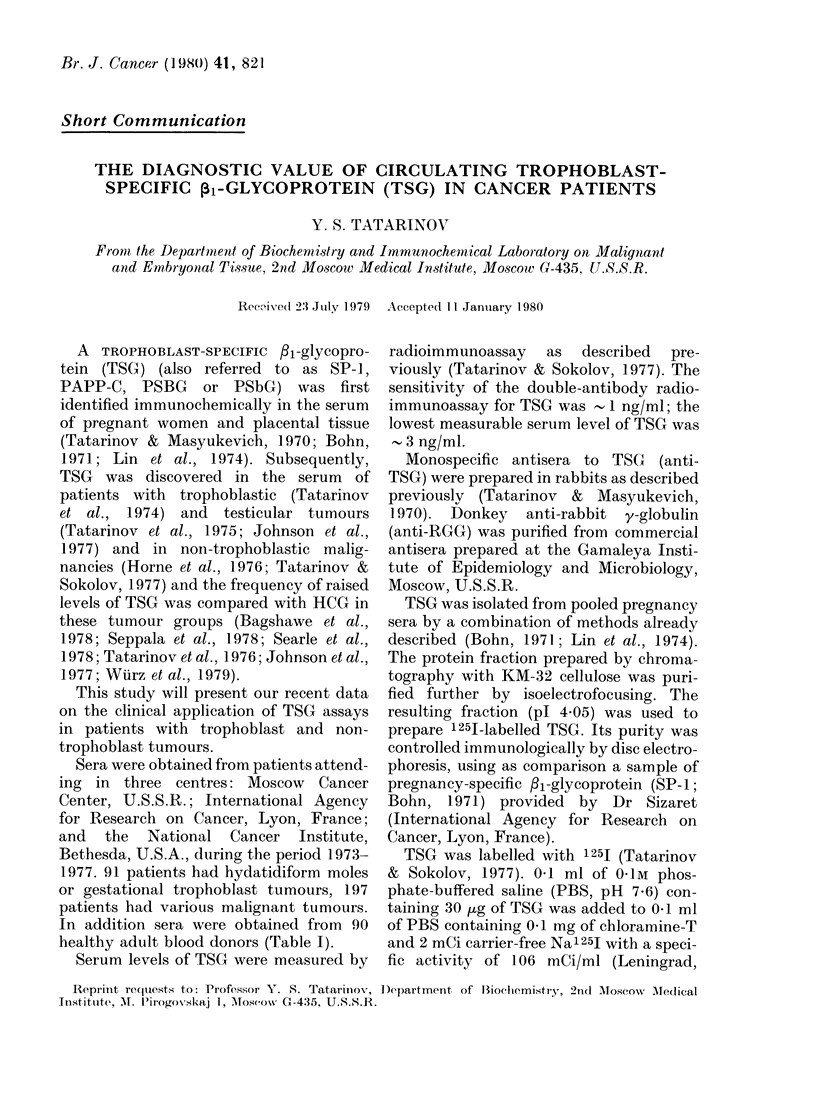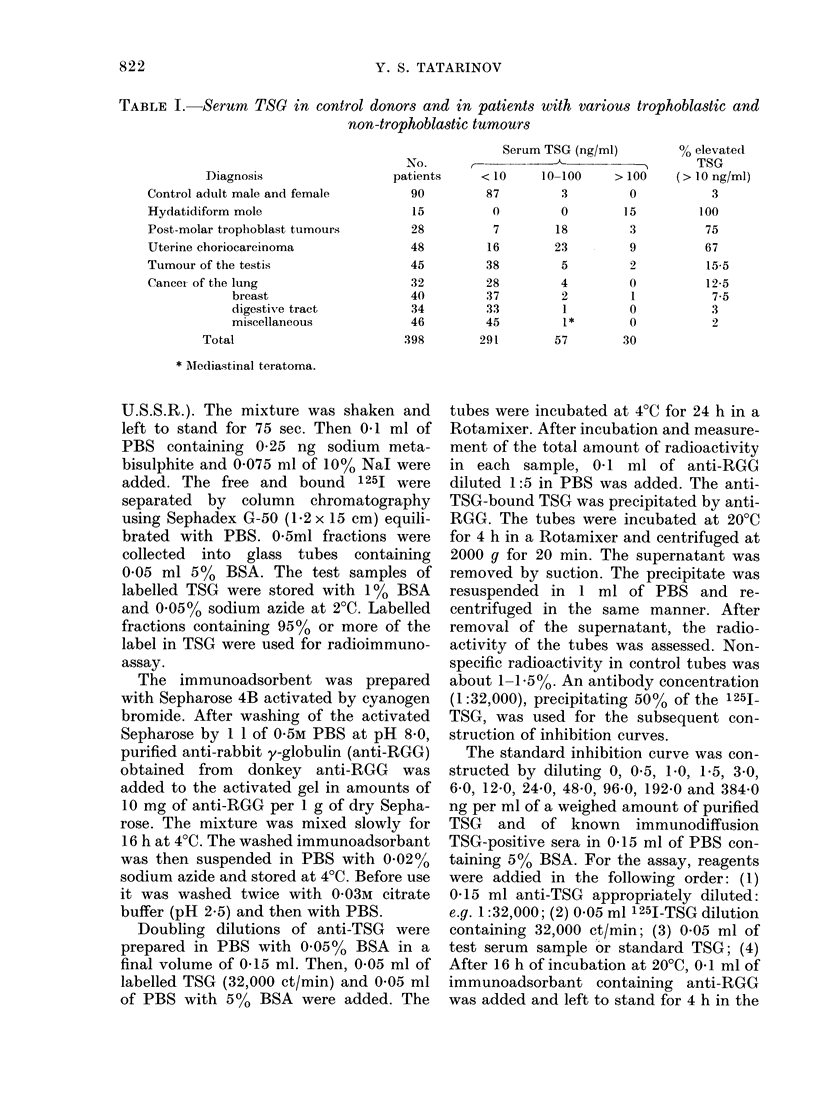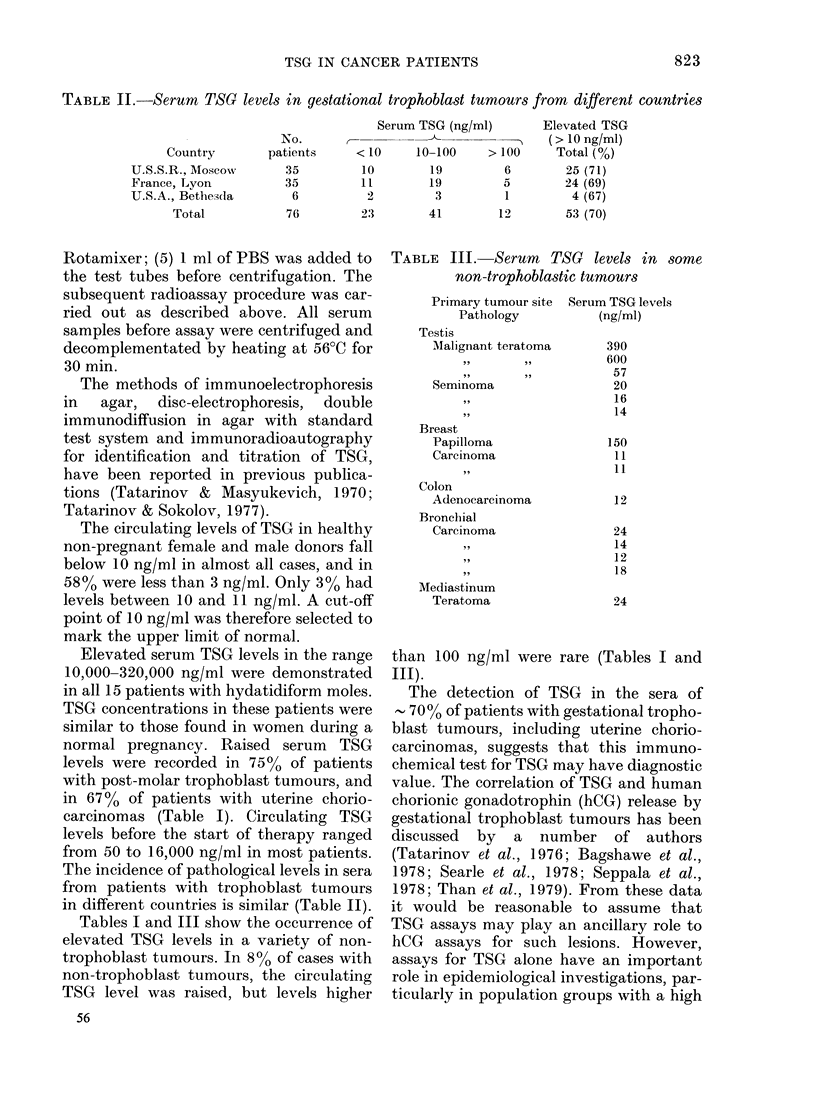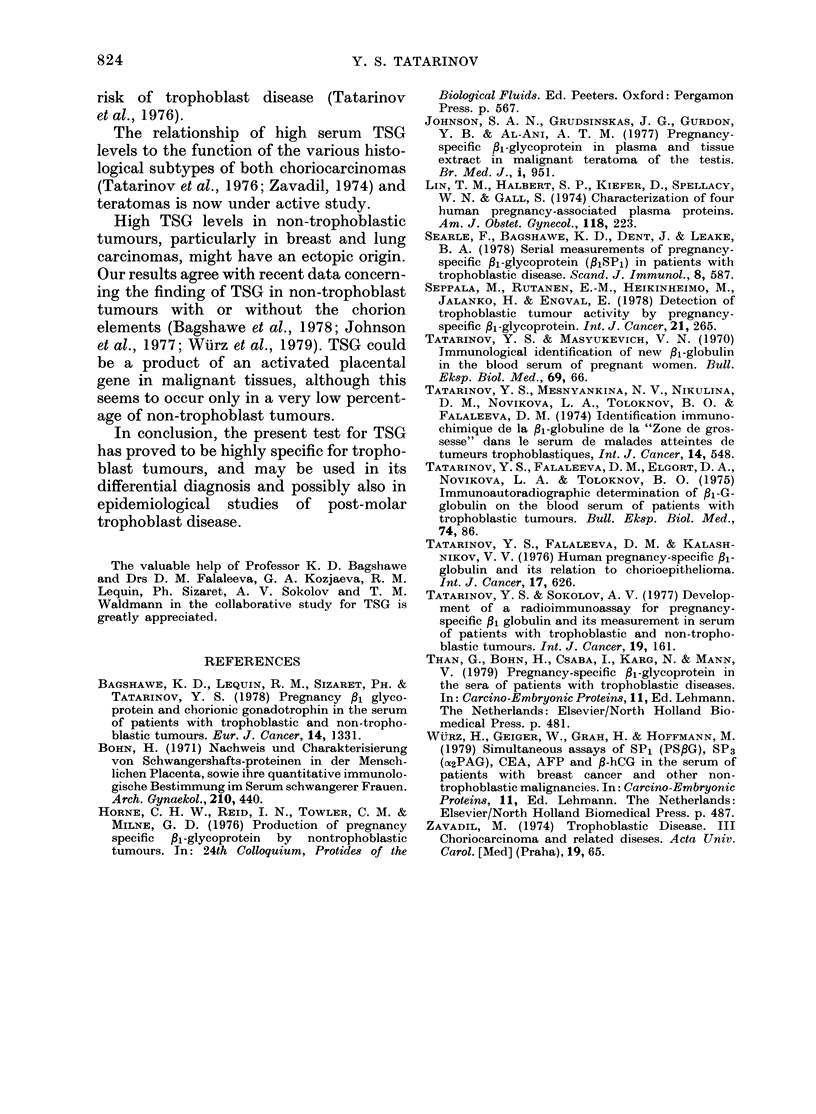# The diagnostic value of circulating trophoblast-specific beta 1-glycoprotein (TSG) in cancer patients.

**DOI:** 10.1038/bjc.1980.147

**Published:** 1980-05

**Authors:** Y. S. Tatarinov


*Br. J. Cancer* (1980) **41**, 821

***Short Communication***

### THE DIAGNOSTIC VALUE OF CIRCULATING TROPHOBLAST-SPECIFIC $\beta_1$-GLYCOPROTEIN (TSG) IN CANCER PATIENTS

Y. S. TATARINOV

*From the Department of Biochemistry and Immunochemical Laboratory on Malignant and Embryonal Tissue, 2nd Moscow Medical Institute, Moscow G-435, U.S.S.R.*

Received 23 July 1979 Accepted 11 January 1980

A TROPHOBLAST-SPECIFIC $\beta_1$-glycoprotein (TSG) (also referred to as SP-1, PAPP-C, PSBG or PSbG) was first identified immunochemically in the serum of pregnant women and placental tissue (Tatarinov & Masyukevich, 1970; Bohn, 1971; Lin *et al.*, 1974). Subsequently, TSG was discovered in the serum of patients with trophoblastic (Tatarinov *et al.*, 1974) and testicular tumours (Tatarinov *et al.*, 1975; Johnson *et al.*, 1977) and in non-trophoblastic malignancies (Horne *et al.*, 1976; Tatarinov & Sokolov, 1977) and the frequency of raised levels of TSG was compared with HCG in these tumour groups (Bagshawe *et al.*, 1978; Seppala *et al.*, 1978; Searle *et al.*, 1978; Tatarinov *et al.*, 1976; Johnson *et al.*, 1977; Würz *et al.*, 1979).

This study will present our recent data on the clinical application of TSG assays in patients with trophoblast and non-trophoblast tumours.

Sera were obtained from patients attending in three centres: Moscow Cancer Center, U.S.S.R.; International Agency for Research on Cancer, Lyon, France; and the National Cancer Institute, Bethesda, U.S.A., during the period 1973–1977. 91 patients had hydatidiform moles or gestational trophoblast tumours, 197 patients had various malignant tumours. In addition sera were obtained from 90 healthy adult blood donors (Table I).

Serum levels of TSG were measured by radioimmunoassay as described previously (Tatarinov & Sokolov, 1977). The sensitivity of the double-antibody radioimmunoassay for TSG was ~1 ng/ml; the lowest measurable serum level of TSG was ~3 ng/ml.

Monospecific antisera to TSG (anti-TSG) were prepared in rabbits as described previously (Tatarinov & Masyukevich, 1970). Donkey anti-rabbit $\gamma$-globulin (anti-RGG) was purified from commercial antisera prepared at the Gamaleya Institute of Epidemiology and Microbiology, Moscow, U.S.S.R.

TSG was isolated from pooled pregnancy sera by a combination of methods already described (Bohn, 1971; Lin *et al.*, 1974). The protein fraction prepared by chromatography with KM-32 cellulose was purified further by isoelectrofocusing. The resulting fraction (pI 4·05) was used to prepare $^{125}$I-labelled TSG. Its purity was controlled immunologically by disc electrophoresis, using as comparison a sample of pregnancy-specific $\beta_1$-glycoprotein (SP-1; Bohn, 1971) provided by Dr Sizaret (International Agency for Research on Cancer, Lyon, France).

TSG was labelled with $^{125}$I (Tatarinov & Sokolov, 1977). 0·1 ml of 0·1M phosphate-buffered saline (PBS, pH 7·6) containing 30 $\mu$g of TSG was added to 0·1 ml of PBS containing 0·1 mg of chloramine-T and 2 mCi carrier-free Na$^{125}$I with a specific activity of 106 mCi/ml (Leningrad,

Reprint requests to: Professor Y. S. Tatarinov, Department of Biochemistry, 2nd Moscow Medical Institute, M. Pirogovskaj 1, Moscow G-435, U.S.S.R.

TABLE I.—*Serum TSG in control donors and in patients with various trophoblastic and non-trophoblastic tumours*

| Diagnosis | No. patients | Serum TSG (ng/ml) < 10 | 10–100 | > 100 | % elevated TSG (> 10 ng/ml) |
|---|---|---|---|---|---|
| Control adult male and female | 90 | 87 | 3 | 0 | 3 |
| Hydatidiform mole | 15 | 0 | 0 | 15 | 100 |
| Post-molar trophoblast tumours | 28 | 7 | 18 | 3 | 75 |
| Uterine choriocarcinoma | 48 | 16 | 23 | 9 | 67 |
| Tumour of the testis | 45 | 38 | 5 | 2 | 15·5 |
| Cancer of the lung | 32 | 28 | 4 | 0 | 12·5 |
| breast | 40 | 37 | 2 | 1 | 7·5 |
| digestive tract | 34 | 33 | 1 | 0 | 3 |
| miscellaneous | 46 | 45 | 1* | 0 | 2 |
| Total | 398 | 291 | 57 | 30 | |

* Mediastinal teratoma.

U.S.S.R.). The mixture was shaken and left to stand for 75 sec. Then 0·1 ml of PBS containing 0·25 ng sodium metabisulphite and 0·075 ml of 10% NaI were added. The free and bound $^{125}$I were separated by column chromatography using Sephadex G-50 (1·2 × 15 cm) equilibrated with PBS. 0·5ml fractions were collected into glass tubes containing 0·05 ml 5% BSA. The test samples of labelled TSG were stored with 1% BSA and 0·05% sodium azide at 2°C. Labelled fractions containing 95% or more of the label in TSG were used for radioimmunoassay.

The immunoadsorbent was prepared with Sepharose 4B activated by cyanogen bromide. After washing of the activated Sepharose by 1 l of 0·5M PBS at pH 8·0, purified anti-rabbit γ-globulin (anti-RGG) obtained from donkey anti-RGG was added to the activated gel in amounts of 10 mg of anti-RGG per 1 g of dry Sepharose. The mixture was mixed slowly for 16 h at 4°C. The washed immunoadsorbant was then suspended in PBS with 0·02% sodium azide and stored at 4°C. Before use it was washed twice with 0·03M citrate buffer (pH 2·5) and then with PBS.

Doubling dilutions of anti-TSG were prepared in PBS with 0·05% BSA in a final volume of 0·15 ml. Then, 0·05 ml of labelled TSG (32,000 ct/min) and 0·05 ml of PBS with 5% BSA were added. The tubes were incubated at 4°C for 24 h in a Rotamixer. After incubation and measurement of the total amount of radioactivity in each sample, 0·1 ml of anti-RGG diluted 1:5 in PBS was added. The anti-TSG-bound TSG was precipitated by anti-RGG. The tubes were incubated at 20°C for 4 h in a Rotamixer and centrifuged at 2000 *g* for 20 min. The supernatant was removed by suction. The precipitate was resuspended in 1 ml of PBS and re-centrifuged in the same manner. After removal of the supernatant, the radioactivity of the tubes was assessed. Non-specific radioactivity in control tubes was about 1–1·5%. An antibody concentration (1:32,000), precipitating 50% of the $^{125}$I-TSG, was used for the subsequent construction of inhibition curves.

The standard inhibition curve was constructed by diluting 0, 0·5, 1·0, 1·5, 3·0, 6·0, 12·0, 24·0, 48·0, 96·0, 192·0 and 384·0 ng per ml of a weighed amount of purified TSG and of known immunodiffusion TSG-positive sera in 0·15 ml of PBS containing 5% BSA. For the assay, reagents were addied in the following order: (1) 0·15 ml anti-TSG appropriately diluted: *e.g.* 1:32,000; (2) 0·05 ml $^{125}$I-TSG dilution containing 32,000 ct/min; (3) 0·05 ml of test serum sample or standard TSG; (4) After 16 h of incubation at 20°C, 0·1 ml of immunoadsorbant containing anti-RGG was added and left to stand for 4 h in the

TABLE II.—*Serum TSG levels in gestational trophoblast tumours from different countries*

| Country | No. patients | Serum TSG (ng/ml) < 10 | 10–100 | > 100 | Elevated TSG (> 10 ng/ml) Total (%) |
|---|---|---|---|---|---|
| U.S.S.R., Moscow | 35 | 10 | 19 | 6 | 25 (71) |
| France, Lyon | 35 | 11 | 19 | 5 | 24 (69) |
| U.S.A., Bethesda | 6 | 2 | 3 | 1 | 4 (67) |
| Total | 76 | 23 | 41 | 12 | 53 (70) |

Rotamixer; (5) 1 ml of PBS was added to the test tubes before centrifugation. The subsequent radioassay procedure was carried out as described above. All serum samples before assay were centrifuged and decomplementated by heating at 56°C for 30 min.

The methods of immunoelectrophoresis in agar, disc-electrophoresis, double immunodiffusion in agar with standard test system and immunoradioautography for identification and titration of TSG, have been reported in previous publications (Tatarinov & Masyukevich, 1970; Tatarinov & Sokolov, 1977).

The circulating levels of TSG in healthy non-pregnant female and male donors fall below 10 ng/ml in almost all cases, and in 58% were less than 3 ng/ml. Only 3% had levels between 10 and 11 ng/ml. A cut-off point of 10 ng/ml was therefore selected to mark the upper limit of normal.

Elevated serum TSG levels in the range 10,000–320,000 ng/ml were demonstrated in all 15 patients with hydatidiform moles. TSG concentrations in these patients were similar to those found in women during a normal pregnancy. Raised serum TSG levels were recorded in 75% of patients with post-molar trophoblast tumours, and in 67% of patients with uterine choriocarcinomas (Table I). Circulating TSG levels before the start of therapy ranged from 50 to 16,000 ng/ml in most patients. The incidence of pathological levels in sera from patients with trophoblast tumours in different countries is similar (Table II).

Tables I and III show the occurrence of elevated TSG levels in a variety of non-trophoblast tumours. In 8% of cases with non-trophoblast tumours, the circulating TSG level was raised, but levels higher than 100 ng/ml were rare (Tables I and III).

TABLE III.—*Serum TSG levels in some non-trophoblastic tumours*

| Primary tumour site Pathology | Serum TSG levels (ng/ml) |
|---|---|
| Testis | |
| Malignant teratoma | 390 |
| ,, ,, | 600 |
| ,, ,, | 57 |
| Seminoma | 20 |
| ,, | 16 |
| ,, | 14 |
| Breast | |
| Papilloma | 150 |
| Carcinoma | 11 |
| ,, | 11 |
| Colon | |
| Adenocarcinoma | 12 |
| Bronchial | |
| Carcinoma | 24 |
| ,, | 14 |
| ,, | 12 |
| ,, | 18 |
| Mediastinum | |
| Teratoma | 24 |

The detection of TSG in the sera of ~ 70% of patients with gestational trophoblast tumours, including uterine choriocarcinomas, suggests that this immunochemical test for TSG may have diagnostic value. The correlation of TSG and human chorionic gonadotrophin (hCG) release by gestational trophoblast tumours has been discussed by a number of authors (Tatarinov *et al.*, 1976; Bagshawe *et al.*, 1978; Searle *et al.*, 1978; Seppala *et al.*, 1978; Than *et al.*, 1979). From these data it would be reasonable to assume that TSG assays may play an ancillary role to hCG assays for such lesions. However, assays for TSG alone have an important role in epidemiological investigations, particularly in population groups with a high risk of trophoblast disease (Tatarinov *et al.*, 1976).

The relationship of high serum TSG levels to the function of the various histological subtypes of both choriocarcinomas (Tatarinov *et al.*, 1976; Zavadil, 1974) and teratomas is now under active study.

High TSG levels in non-trophoblastic tumours, particularly in breast and lung carcinomas, might have an ectopic origin. Our results agree with recent data concerning the finding of TSG in non-trophoblast tumours with or without the chorion elements (Bagshawe *et al.*, 1978; Johnson *et al.*, 1977; Würz *et al.*, 1979). TSG could be a product of an activated placental gene in malignant tissues, although this seems to occur only in a very low percentage of non-trophoblast tumours.

In conclusion, the present test for TSG has proved to be highly specific for trophoblast tumours, and may be used in its differential diagnosis and possibly also in epidemiological studies of post-molar trophoblast disease.

The valuable help of Professor K. D. Bagshawe and Drs D. M. Falaleeva, G. A. Kozjaeva, R. M. Lequin, Ph. Sizaret, A. V. Sokolov and T. M. Waldmann in the collaborative study for TSG is greatly appreciated.

### REFERENCES

BAGSHAWE, K. D., LEQUIN, R. M., SIZARET, PH. & TATARINOV, Y. S. (1978) Pregnancy $\beta_1$ glycoprotein and chorionic gonadotrophin in the serum of patients with trophoblastic and non-trophoblastic tumours. *Eur. J. Cancer*, **14**, 1331.

BOHN, H. (1971) Nachweis und Charakterisierung von Schwangershafts-proteinen in der Menschlichen Placenta, sowie ihre quantitative immunologische Bestimmung im Serum schwangerer Frauen. *Arch. Gynaekol.*, **210**, 440.

HORNE, C. H. W., REID, I. N., TOWLER, C. M. & MILNE, G. D. (1976) Production of pregnancy specific $\beta_1$-glycoprotein by nontrophoblastic tumours. In: *24th Colloquium, Protides of the Biological Fluids*. Ed. Peeters. Oxford: Pergamon Press. p. 567.

JOHNSON, S. A. N., GRUDSINSKAS, J. G., GURDON, Y. B. & AL-ANI, A. T. M. (1977) Pregnancy-specific $\beta_1$-glycoprotein in plasma and tissue extract in malignant teratoma of the testis. *Br. Med. J.*, **i**, 951.

LIN, T. M., HALBERT, S. P., KIEFER, D., SPELLACY, W. N. & GALL, S. (1974) Characterization of four human pregnancy-associated plasma proteins. *Am. J. Obstet. Gynecol.*, **118**, 223.

SEARLE, F., BAGSHAWE, K. D., DENT, J. & LEAKE, B. A. (1978) Serial measurements of pregnancy-specific $\beta_1$-glycoprotein ($\beta_1SP_1$) in patients with trophoblastic disease. *Scand. J. Immunol.*, **8**, 587.

SEPPALA, M., RUTANEN, E.-M., HEIKINHEIMO, M., JALANKO, H. & ENGVAL, E. (1978) Detection of trophoblastic tumour activity by pregnancy-specific $\beta_1$-glycoprotein. *Int. J. Cancer*, **21**, 265.

TATARINOV, Y. S. & MASYUKEVICH, V. N. (1970) Immunological identification of new $\beta_1$-globulin in the blood serum of pregnant women. *Bull. Eksp. Biol. Med.*, **69**, 66.

TATARINOV, Y. S., MESNYANKINA, N. V., NIKULINA, D. M., NOVIKOVA, L. A., TOLOKNOV, B. O. & FALALEEVA, D. M. (1974) Identification immunochimique de la $\beta_1$-globuline de la "Zone de grossesse" dans le serum de malades atteintes de tumeurs trophoblastiques, *Int. J. Cancer*, **14**, 548.

TATARINOV, Y. S., FALALEEVA, D. M., ELGORT, D. A., NOVIKOVA, L. A. & TOLOKNOV, B. O. (1975) Immunoautoradiographic determination of $\beta_1$-G-globulin on the blood serum of patients with trophoblastic tumours. *Bull. Eksp. Biol. Med.*, **74**, 86.

TATARINOV, Y. S., FALALEEVA, D. M. & KALASHNIKOV, V. V. (1976) Human pregnancy-specific $\beta_1$-globulin and its relation to chorioepithelioma. *Int. J. Cancer*, **17**, 626.

TATARINOV, Y. S. & SOKOLOV, A. V. (1977) Development of a radioimmunoassay for pregnancy-specific $\beta_1$ globulin and its measurement in serum of patients with trophoblastic and non-trophoblastic tumours. *Int. J. Cancer*, **19**, 161.

THAN, G., BOHN, H., CSABA, I., KARG, N. & MANN, V. (1979) Pregnancy-specific $\beta_1$-glycoprotein in the sera of patients with trophoblastic diseases. In: *Carcino-Embryonic Proteins*, **11**, Ed. Lehmann. The Netherlands: Elsevier/North Holland Biomedical Press. p. 481.

WÜRZ, H., GEIGER, W., GRAH, H. & HOFFMANN, M. (1979) Simultaneous assays of $SP_1$ ($PS\beta G$), $SP_3$ ($\alpha_2PAG$), CEA, AFP and $\beta$-hCG in the serum of patients with breast cancer and other non-trophoblastic malignancies. In: *Carcino-Embryonic Proteins*, **11**, Ed. Lehmann. The Netherlands: Elsevier/North Holland Biomedical Press. p. 487.

ZAVADIL, M. (1974) Trophoblastic Disease. III Choriocarcinoma and related diseses. *Acta Univ. Carol.* [Med] (Praha), **19**, 65.